# 3D Printed e-Tongue

**DOI:** 10.3389/fchem.2018.00151

**Published:** 2018-05-03

**Authors:** Gabriel Gaál, Tatiana A. da Silva, Vladimir Gaál, Rafael C. Hensel, Lucas R. Amaral, Varlei Rodrigues, Antonio Riul

**Affiliations:** ^1^Applied Physics Department, University of Campinas, Campinas, Brazil; ^2^School of Agricultural Engineering, University of Campinas, Campinas, Brazil

**Keywords:** 3D printing, electronic tongue, interdigitated electrodes, conductive 3D printing filament, soil analysis, soil spectroscopy, precision agriculture

## Abstract

Nowadays, one of the biggest issues addressed to electronic sensor fabrication is the build-up of efficient electrodes as an alternative way to the expensive, complex and multistage processes required by traditional techniques. Printed electronics arises as an interesting alternative to fulfill this task due to the simplicity and speed to stamp electrodes on various surfaces. Within this context, the Fused Deposition Modeling 3D printing is an emerging, cost-effective and alternative technology to fabricate complex structures that potentiates several fields with more creative ideas and new materials for a rapid prototyping of devices. We show here the fabrication of interdigitated electrodes using a standard home-made CoreXY 3D printer using transparent and graphene-based PLA filaments. Macro 3D printed electrodes were easily assembled within 6 min with outstanding reproducibility. The electrodes were also functionalized with different nanostructured thin films via dip-coating Layer-by-Layer technique to develop a 3D printed e-tongue setup. As a proof of concept, the printed e-tongue was applied to soil analysis. A control soil sample was enriched with several macro-nutrients to the plants (N, P, K, S, Mg, and Ca) and the discrimination was done by electrical impedance spectroscopy of water solution of the soil samples. The data was analyzed by Principal Component Analysis and the 3D printed sensor distinguished clearly all enriched samples despite the complexity of the soil chemical composition. The 3D printed e-tongue successfully used in soil analysis encourages further investments in developing new sensory tools for precision agriculture and other fields exploiting the simplicity and flexibility offered by the 3D printing techniques.

## 1. Introduction

The continuous increase for food demand and limited productive crop areas have stimulated the development of new precision agriculture tools to avoid excessive and/or insufficient use of fertilizers and pesticides in soil management. Therefore, high-detailed data for soil characterization is fundamental for a precise soil management. However, current soil chemical analysis protocols are time-consuming and expensive procedures demanding alternative approaches for rapid and on-site soil characterization (Adamchuk and Viscarra Rossel, 2010).

There are basically two approaches for point-of-care soil characterization, one of them based on chemical extraction of specific soil macro-nutrients and then its detection by ion-selective electrodes (ISE) or ion selective field effect transistors (ISFET) (Artigas et al., 2001; Kim et al., 2007; Shaw et al., 2013). Despite the precise quantitative characterization, some parameters need to be tuned for each ion present on the soil sample, making it complicated for measurements of various nutrients or even the use of several instruments. The other approach is the direct measurement of the soil fertility parameters, and the most used techniques are optical spectroscopy (An et al., 2014; Vohland et al., 2014), capillarity electrophoresis (Smolka et al., 2017) and electronic tongues (Mimendia et al., 2014; Braunger et al., 2017). Optical spectroscopy provides several soil properties through rapid and simple measurements, but the lack of correlation between spectral bands and concentration of soil nutrients leads to models with high prediction errors. Electrophoresis is an interesting approach as it uses high electrical potential in order to separate ions based on their net charge. It provides accurate qualitative measurements of certain ions presented on samples, and also quantitative estimations of their concentrations. However, the need of high electrical potentials (~2000 V) may hamper some on-site applications. On the other hand, electronic tongue (e-tongue) sensors detect variations of the analyte dielectric constant, ensuring high sensitivity with no need of specific interactions (Riul et al., 2010). They have been widely used in quality control of foodstuff, beverages and pharmaceuticals, in addition to clinical and environmental analysis. Moreover, e-tongue devices provide rapid and continuous analysis of complex systems and fast experiments for either qualitative, semi-quantitative or quantitative analyses (Legin et al., 2003; Citterio and Suzuki, 2008; Shimizu et al., 2017).

One kind of e-tongue sensor uses interdigitated electrodes (IDEs) which are arrays of parallel plates capacitors in order to measure the analyte dielectric constant. The IDE geometry maximizes the capacitor effective area and then it increases its overall sensitivity (Olthuis et al., 1995; Igreja and Dias, 2011). The electrode fabrication usually involves expensive, complex and multistage micro-fabrication processes which still involves the use of toxic reagents. In that sense, there are great efforts to develop alternative techniques such as ink-jet printing, screen-printing and direct drawing processes exploiting conductive inks for electrode fabrication (Tomazelli Coltro et al., 2004; Coltro et al., 2010; Cummins and Desmulliez, 2012; Nakashima et al., 2012; Perinka et al., 2013; Chagas et al., 2015; Paula et al., 2018). However, these techniques still requires further steps to integrate the electrodes to assemble a functional device. The use of 3D printers in such task permits an easy integration of the electrodes to complex and intricate 3D structures in order to build up sensors in a straightforward manner (Gaal et al., 2017). Recent advances in thermoplastic materials used as filaments for Fused Deposition Modeling (FDM) 3D printing allowed an easy fabrication of 3D printed electrodes using a commercial conductive filament for electrochemical applications (Foster et al., 2017).

In this work we aim the development of planar 3D printed IDEs in order to assemble a proof of concept e-tongue. We have exploited this simple 3D-printed e-tongue sensor to discriminate soil samples enriched with important nutrients for crop production. Planar IDEs were printed in a home-made FDM 3D printer, being further functionalized with nanostrucutred Layer-by-Layer (LbL) films to be used as sensing units (Riul et al., 2003). Soil samples diluted in ultra-pure water were characterized via Principal Component Analysis (PCA) of the Electrical Impedance Spectroscopy (EIS) and a clear distinction among all samples was obtained.

## 2. Materials and methods

### 2.1. 3D printer

**Figure 1:**
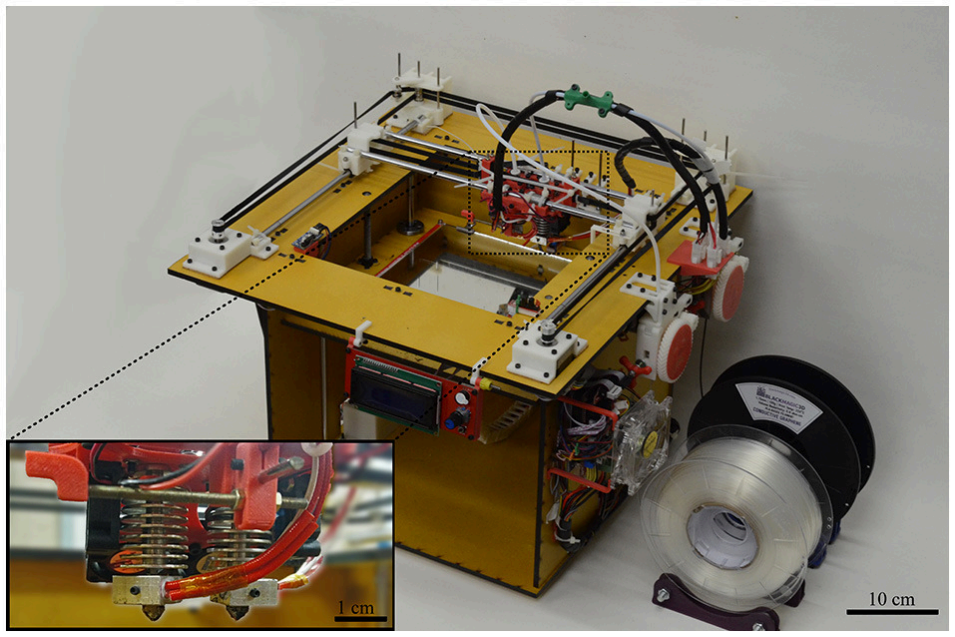
Two noozle home-made CoreXY 3D printer used to produce the planar IDEs. Inset: 0.4 mm in diameter two nozzles setup.

A two nozzle home-made CoreXY FDM 3D printer was built based on the RepRap open hardware, displayed in Figure 1. It uses two commercial hot nozzles of 400 μm in diameter to extrude thermoplastics filaments of 1.75 mm in diameter. The molten filament is deposited over a hot bed to ensure a good adhesion of the first layer and to maintain a constant temperature gradient along the printed layers avoiding delamination. The hot bed is formed by a commercial heated plate coupled with a mirror, which provides a smooth and flat printing surface with building area of 200 mm × 200 mm. The printing heads are moved in the XY plane by two stepper motors following the H-frame type XY-positioning system (Itoh et al., 2004; Sollmann et al., 2010) and the hot bed is moved in the z-axis by another couple of stepper motors. The printer control is done by an open source Arduino microcontroller board Mega 2560, interfaced with a commercial RepRap Arduino Mega Pololu Shield (RAMPS).

The design of the interdigitated electrodes were done with Autodesk Inventor 2015 Student Edition and were further converted to STereoLithography (STL) format. The STL models were sliced using the free license software Slic3r optimized for two extruders. The slicing procedure converts the STL 3D models and transforms them into stacks of 2D printing planes, further interpreted by the printer hardware.

**Figure 2:**
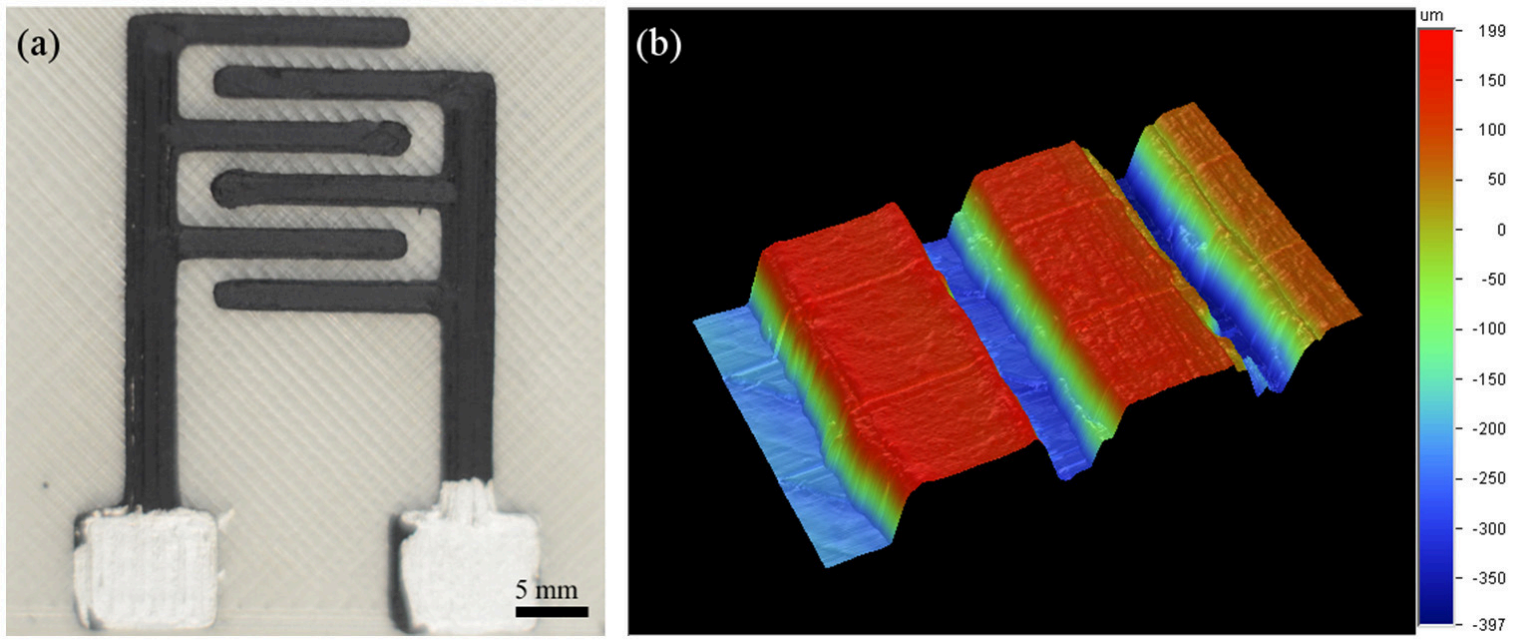
**(a)** 3D printed IDE with 2 mm finger thick and 1.4 mm of finger separation. **(b)** 3D profilometry mapping of a printed IDE.

Planar IDEs, Figure 2a of several geometries were 3D-printed in order to verify the limits of our system and demonstrate the facility to tune the device geometry. For the e-tongue system planar IDEs were designed to have 3 pairs of fingers 9 mm long, 1 mm width and 1 mm spaced each other. The IDE base was comprised of 2 planes having 0.4 mm thickness that were printed with transparent Poly Lactic Acid (PLA) purchased from e3D. The conductive filament is a commercial PLA-based thermoplastic doped with graphene fibers, purchased from BlackMagic 3D. A profilometer Dektak 150 was used to estimate the root-mean-square (RMS) surface roughness of the printed conductive tracks.

#### 2.1.1. Chemical treatment

Before the dip-coating deposition, it was applied a chemical treatment on the surface of the printed IDEs in order to ensure the adhesion of the polyelectrolytes. The printed IDEs were placed in a solution of KMnO_4_-H_2_SO_4_ (50 mL), prepared from KMnO_4_ (194 mg) dissolved in H_2_SO_4_ (1 M). They were maintained in ultrasonic bath for 3 hours, and then washed with 1 L of ultra-pure water and HCl (1 M) (Martins et al., 2014). To remove the MnO_2_ adhered to the surface of the IDEs a further cleaning process was performed with a 1 M H_2_SO_4_ (25%) solution, and a 30% H_2_O_2_ (75%) solution. Ultra-pure water was provided from an Arium Comfort Sartorius system that was used also to prepare all polyelectrolyte solutions described below.

#### 2.1.2. LbL dip-coating

The LbL polymer film deposition was made in a home-made setup based on an Arduino board UNO and stepper motors. This setup allows a fully automated LbL film mounting with precise control of a large number of parameters for the LbL film deposition such as dipping velocities, time of immersion in each polyelectrolyte, wash and dry times (Hensel et al., 2018).

It was used three different LbL films in which the anionic layers used were copper phthalocyanine-3,4′,4′′,4‴-tetrasulfonic acid tetrasodium salt (CuTsPc), montmorillonite K (MMt-K), poly(3,4-ethylenedioxythiophene)-poly(styrenesulfonate) (PEDOT:PSS), and for all three LbL architectures poly(diallyldimethylammonium chloride) (PDDA) was used as the cationic layer. The aqueous CuTsPc solution was used at 0.5 mg/mL and pH 8, the MMt-K water solution was used at 1 mg/mL and pH 3, and the PEDOT:PSS solution was used at 0.2 mg/mL and pH 3. The cationic PDDA solution was prepared at 10 μL/mL and the pH was adjusted to be the same as the corresponding anionic polyelectrolyte forming the LbL film. The immersion time was 10 min for both anionic and cationic layers, and it was kept the same for all films deposited. 50 bilayers were deposited on each 3D-printed IDE and the LbL deposition was confirmed by the difference of the coated IDE impedance spectrum in air compared with that of the bare electrode also in air.

### 2.2. Electronic tongue

The e-tongue sensor was comprised of 4 sensing units, one bare IDE and three coated with nanostructured films described above. The sensor is based on the impedance measurement of the IDEs immersed in the liquid system, comparing the electrical response at a fixed frequency of different samples via Principal Component Analysis (PCA). Briefly, PCA is a multivariate statistical tool that reduces the dimensionality of the original data set facilitating correlation and visualization. This procedure is based on a linear transformation that maximizes the variance of the initial matrix and plot the new data on a new set of orthogonal axis called Principal Components without losing information (Rencher, 2012).

#### 2.2.1. Soil samples

The soil samples were extracted from the same location and separated into seven pots with 1 L capacity. Each of them was added NH_4_NO_3_, NH_4_H_2_PO_4_, KCl, CaCl_2_(H_2_O)_2_, MgCl_2_(H_2_O)_6_, or (NH_4_)_2_SO_4_, in order to separately fertilize six soil samples with nitrogen (N), phosphorus (P), potassium (K), calcium (Ca), magnesium (Mg) or sulfur (S), respectively. A seventh sample was kept unfertilized as the control. All pots were maintained for 40 days in a greenhouse with daily irrigation to allow chemical reactions and full fertilization of the soils. To quantify the amount of macro-nutrients available to the plants, a portion of the samples was sent to a commercial laboratory for traditional chemical analysis.

The samples used for the 3D printed e-tongue analysis were diluted in 25 mL of ultra-pure water at 1 mg/mL. It was used a commercial Frequency Response Analyzer (FRA) Solartron 1260A with a Dielectric Interface 1269A to acquire the impedance spectra in ambient conditions. The data was analyzed at 1 kHz, as at the kHz frequency region the impedance of the system is known to be dominated by the film/electrode interface (Riul et al., 2003). The impedance spectrum was acquired for each soil sample and after the measurement the IDEs were thoroughly washed in ultra-pure water. A control EIS was then performed in ultra-pure water to verify cross-contamination of the electrodes.

## 3. Results and discussion

### 3.1. 3D printed IDEs

An iterative process was used to optimize the printer parameters in order to print different planar IDEs. Figure 2a illustrates a particular IDE configuration that were printed within less than 10 min. It is worth mentioning that the geometry can be easily modified by simply changing the computational 3D model design, thus facilitating the prototyping process. Profilometry of a printed IDE finger showed a 6 μm RMS surface roughness on a square region on the top of the printed conductive track, Figure 2b.

**Figure 3:**
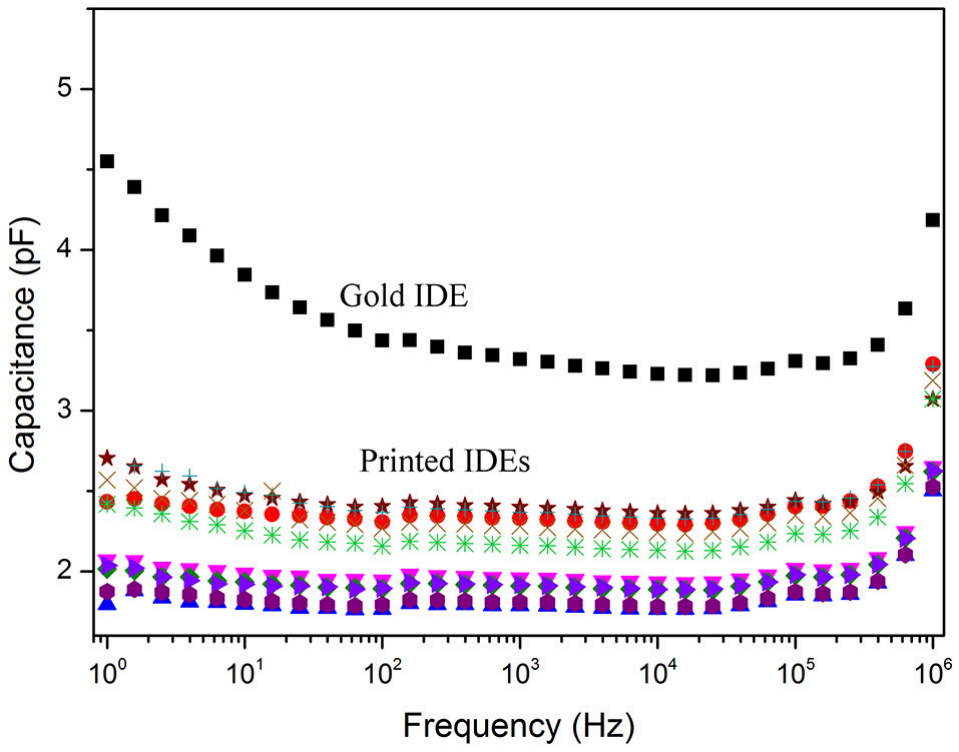
Capacitance spectra of a gold IDE and 10 different 3D printed electrodes.

Figure 3 illustrates the capacitance response in air among ten different 3D printed IDEs when compared with a gold IDE onto glass substrate with similar geometric parameters. As expected, the frequency response of the printed IDEs is analogous to the gold electrode. Moreover, besides the rapid prototyping offered by the 3D-printing technique it was observed an outstanding reproducibility of the geometric parameters of the printed devices as their capacitance spectra deviated in 1 pF range.

### 3.2. LbL deposition

**Figure 4:**
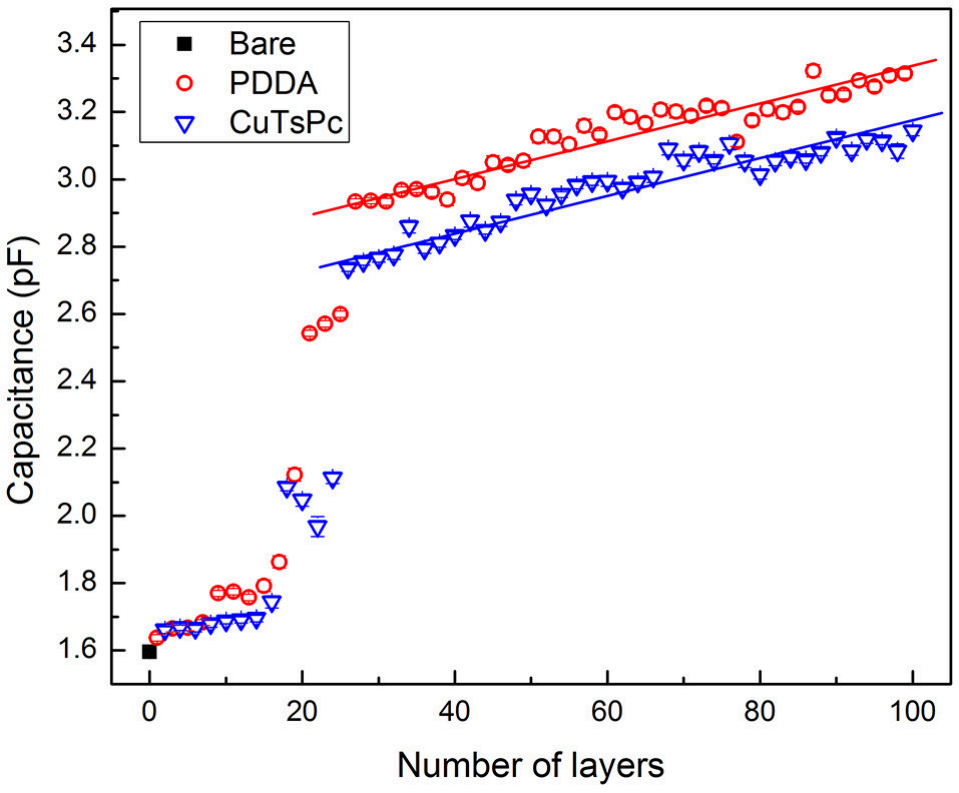
Capacitance measurements in air at 1 kHz as function of the number of layers deposited for a (PDDA/CuTsPc)_50_ film grown onto a 3D printed IDE.

In order to verify the layer-by-layer growth of the nanostructured films onto the polymeric substrate, the capacitance at 1 kHz was measured in air after each deposition step, Figure 4. Firstly, it was observed an initial non-linear trend in the measured capacitance between two different polyelectrolytes, attributed to the starting adsorption process of materials on the plastic electrodes in the LbL film build-up (Poghossian et al., 2006, 2013; Daikuzono et al., 2015). A linear trend growth was observed only after the twentieth five deposited layer, thus indicating a homogeneous adsorption process of materials on the electrode interface. The (PDDA/CuTsPc)_50_ film deposition was chosen to such analysis as it is easier to compare with recent ongoing studies on the capacitance change in the LbL dipping process made in our research group (Ferreira, 2016; Hensel et al., 2018).

**Figure 5:**
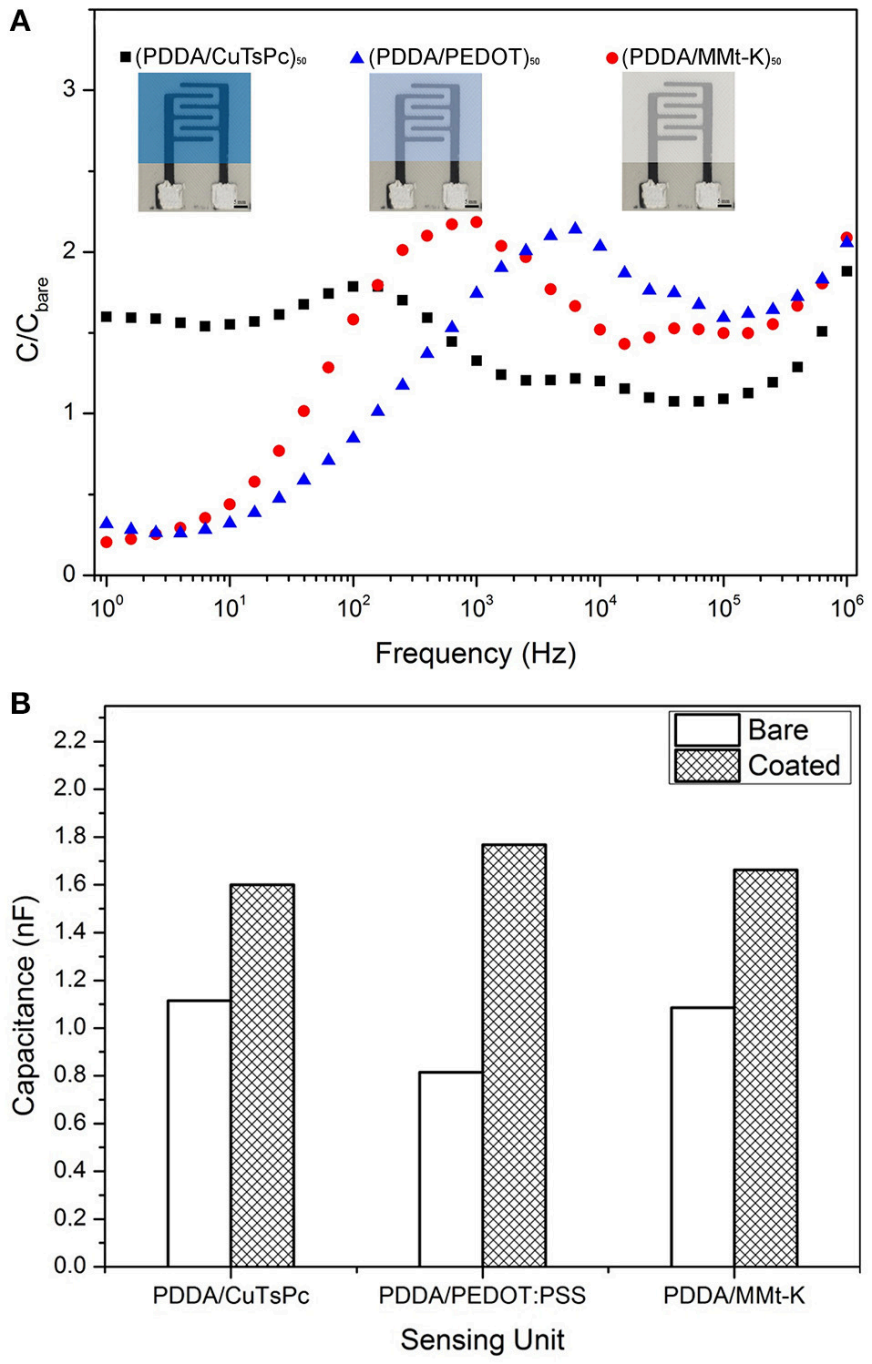
**(A)** Ratio of the coated IDE capacitance spectrum in ultra-pure water to bare IDE response. The bar scale in the IDEs picture is 5 mm. **(B)** Comparison of the capacitance value at 1 kHz of the bare IDE and the coated electrode measured in ultra-pure water.

The final deposition process was confirmed by the difference between the impedance spectrum of the bare electrode and the coated IDE. Figure 5A illustrates the ratio of the real capacitance spectrum of a coated IDE to a bare IDE measured in ultra-pure water. Such graph indicates a change of the capacitive response of the coated IDE in comparison with the bare electrode in all the spectrum range. In particular, Figure 5B shows the comparison of the real capacitance values of the bare IDE and the coated electrodes at 1 kHz measured in ultra-pure water. As discussed previously, this frequency has a major contribution from the film/electrolyte interface (Riul et al., 2003), rendering easy the verification of the presence of thin films onto the printed IDEs.

### 3.3. e-Tongue analysis of soil samples

**Figure 6:**
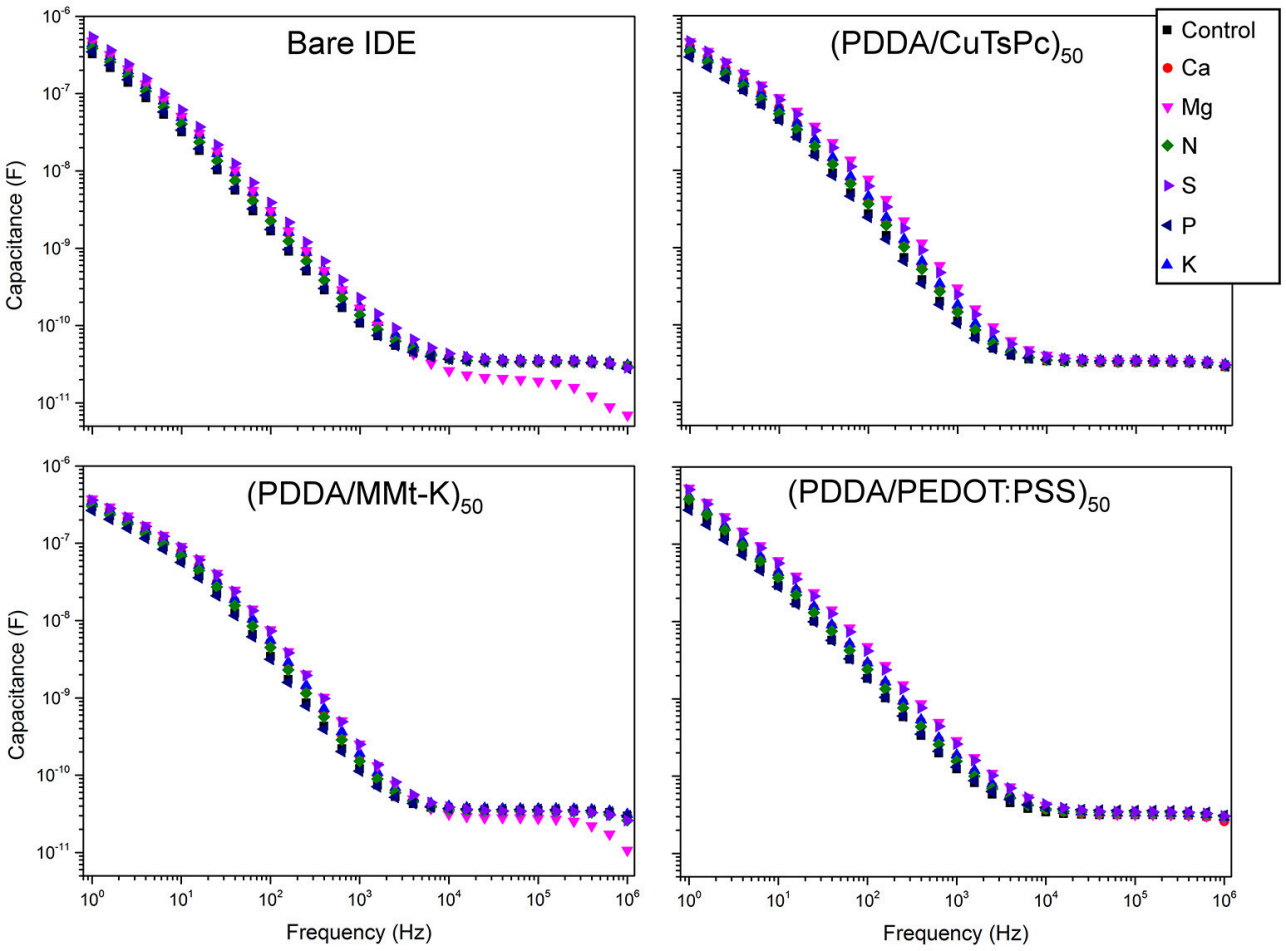
Capacitance spectra of each macro-nutrient solution of all four sensing units.

**Figure 7:**
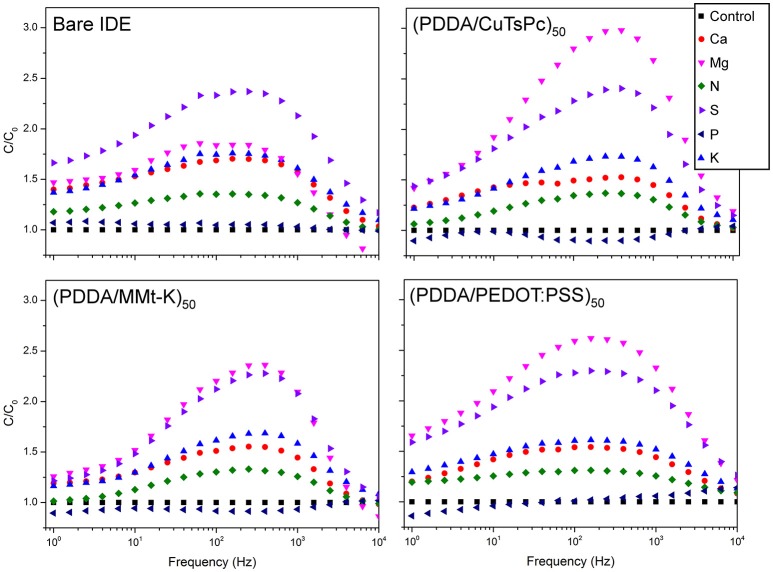
Ratio of the capacitance spectrum of each macro-nutrient solution (C) to the spectrum of the control sample (C_0_) of all the four sensing units.

Figure 6 shows the frequency response of the real capacitance of a single measurement for each soil sample and each sensing unit. As expected at mid to low frequency region (10^4^ Hz to 1 Hz) it can be observed a dispersion of the samples response, moreover, at this frequency range each sensing unit has a different shape of the spectra. It was also evaluated the relative capacitance spectra, Figure 7 which is the ratio between the real capacitance of the sample enriched with a nutrient (C) to the real capacitance of the control sample (C_0_). This analysis allows one to easily identify samples enriched with Mg and S, presenting good distinction from the control, while the phosphorus sample is grouped quite close to the control. This would be expected, since on tropical and poor soils, practically all the applied P must be retained by soil colloids (adsorbed and unavailable to the plants), which causes the similarity between P and control solutions.

**Figure 8:**
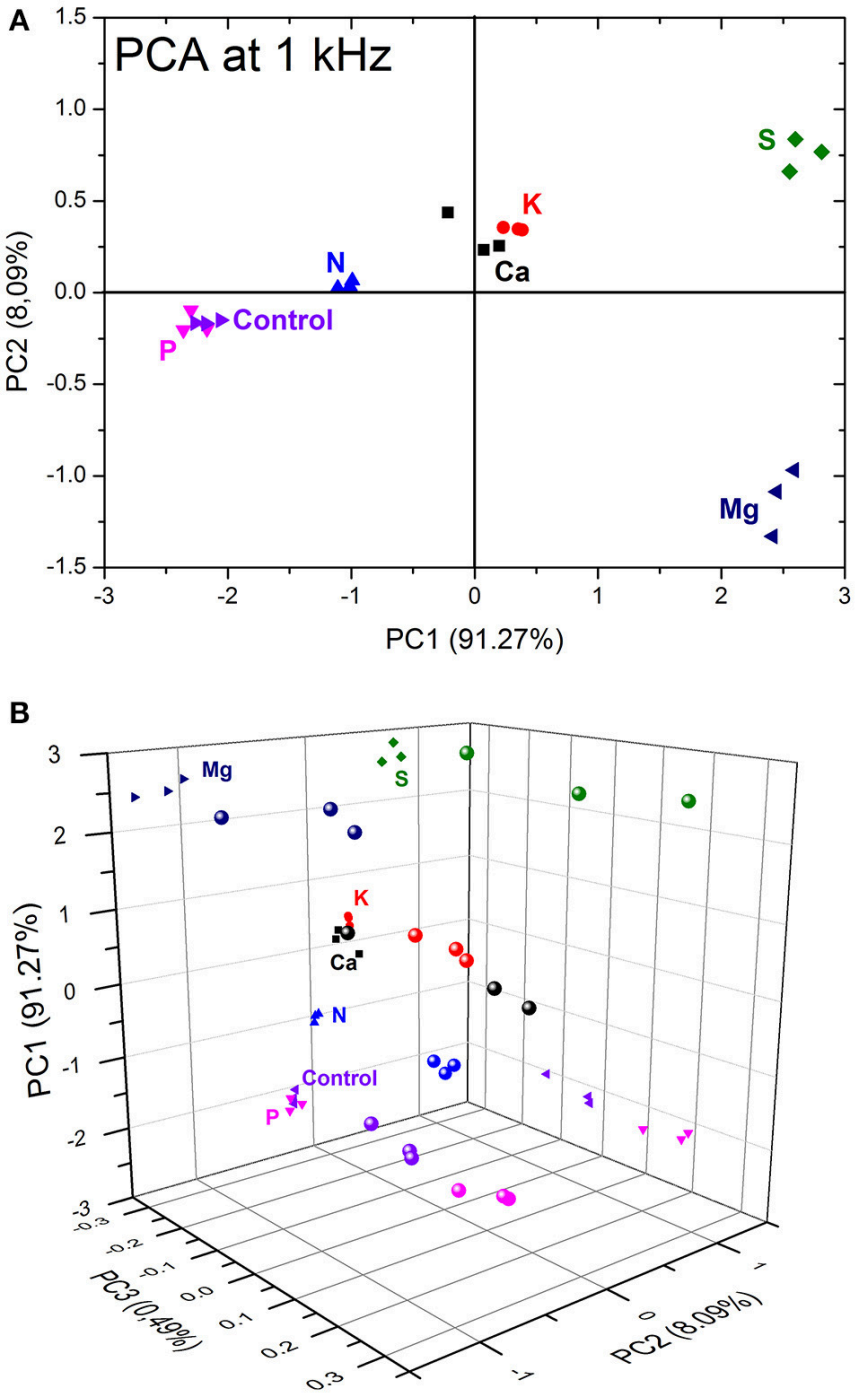
PCA score plot **(A)** 2D PC1 × PC2, and **(B)** 3D plot, evaluated at 1 kHz of the 3D printed e-tongue applied to soil analysis of seven distinct samples.

PCA analysis was applied to a set of three independent real capacitance measurements at 1 kHz from all seven samples, Figure 8A. A good correlation of the rough data and PCA decomposition was observed as the two first principal components add up to 99.36%. Moreover, a good distinction was achieved among the enriched aliquots even considering the high complexity involved in soil analysis. Nevertheless, an expected superposition between the phosphorus and the control samples was observed hindering their distinction as discussed above. One can overcome this superposition considering the third principal component, which accounts for 0.49% of the data variance creating a 3D extension of the PCA plot, Figure 8B. The data projection into the PC3 × PC1 plane shows clearly the separation of the control and phosphorus clusters. Finally, the 2D score plot is often used over the 3D extension because it is easier to see the data clustering, but in some cases it can hide important information and lead to false conclusions.

It is important to stress that this e-tongue system can contribute in the future for point-of-care systems applied in soil analysis and management. There is no need of complicated apparatus as impedance measurements can be taken at a fixed frequency simplifying the development of portable devices. Moreover, statistical analysis that does not require high computational demand can be easily integrated to create a portable tool for soil management. Despite the lack of quantitative information about the soil nutrients, the system can be used as a simplified apparatus to control deviations from the standard soil composition.

## 4. Conclusions

Using a home-made Core-XY FDM 3D printer and a commercial conductive filament, 3D printed IDEs have been successfully fabricated within 6 min with outstanding reproducibility. The electrodes were further functionalized with different nanostructured thin films via dip-coating LbL technique in order to develop a proof of concept 3D printed e-tongue. This system was then applied to soil analysis to discriminate soil aliquots enriched with different macro-nutrients (N, P, K, S, Mg, and Ca). The frequency response of the soil samples diluted in water were verified by electrical impedance spectroscopy, and then compared via PCA analysis. A good distinction of all samples was obtained despite the complexity of the soil chemical composition. Our results show that 3D printing technology potentiates the field of sensor fabrication with cost-effective and alternative materials for a rapid prototyping as well as greater flexibility in design, paving the way for more abundant developments.

## Author contributions

GG: Fabrication of the printed electrodes, growth of the nanostructured LbL films for the e-tongue sensor and PCA analysis of the EIS of the soil samples. TdS: EIS measurements of the soil samples. VG: Project and setup of the Core-XY 3D printer. RH: Growth analysis of the nanostructured polymer films. LA: Preparation of the soil samples, discussions during the writing and revision of the manuscript. VR: Project of the Core-XY 3D printer, discussions during the writing and revision of the manuscript. AR: Principal investigator in this subject.

### Conflict of interest statement

The authors declare that the research was conducted in the absence of any commercial or financial relationships that could be construed as a potential conflict of interest.
